# *secCl* is a cys-loop ion channel necessary for the chloride conductance that mediates hormone-induced fluid secretion in *Drosophila*

**DOI:** 10.1038/s41598-019-42849-9

**Published:** 2019-05-16

**Authors:** Daniel Feingold, Laura Knogler, Tanja Starc, Pierre Drapeau, Michael J. O’Donnell, Laura A. Nilson, Joseph A. Dent

**Affiliations:** 1 Department of Biology, McGill University, 1205 Dr. Penfield, Montréal, Québec, H3A 1B1 Canada; 2 Institute of Neuroscience, Technische Universität München, Biedersteiner Str. 29, München, Bau 601D-80802 Germany; 3 Department of Biology, McMaster University, 1280 Main Street West, Hamilton, Ontario, L8S 4K1 Canada; 4 Department of Neurosciences, Research Centre of the University of Montréal Hospital Centre, Montréal, Québec, Canada; 5 Max Planck Institute of Neurobiology, Sensorimotor Control Research Group, Am Klopferspitz 18, Martinsried, 82152 Germany

**Keywords:** Molecular evolution, Neurophysiology

## Abstract

Organisms use circulating diuretic hormones to control water balance (osmolarity), thereby avoiding dehydration and managing excretion of waste products. The hormones act through G-protein-coupled receptors to activate second messenger systems that in turn control the permeability of secretory epithelia to ions like chloride. In insects, the chloride channel mediating the effects of diuretic hormones was unknown. Surprisingly, we find a pentameric, cys-loop chloride channel, a type of channel normally associated with neurotransmission, mediating hormone-induced transepithelial chloride conductance. This discovery is important because: 1) it describes an unexpected role for pentameric receptors in the membrane permeability of secretory epithelial cells, and 2) it suggests that neurotransmitter-gated ion channels may have evolved from channels involved in secretion.

## Introduction

Organisms have evolved diverse mechanisms to solve the problems of osmoregulation and excretion. In insects, urine is excreted through Malpighian tubules (MTs), the primary renal epithelium. MTs regulate the net flux of ions from the surrounding hemolymph into the tubule lumen, establishing the osmotic gradient that drives fluid secretion. In the prevailing model, cation and anion transport are, for the most part, spatially segregated within the *Drosophila* MT: cation transport is restricted to principal cells and anion transport is primarily restricted to stellate cells^1^,^2^. In principal cells, the apically localized V-type H^+^ ATPase energizes transepithelial secretion, providing electrogenic transport of H^+^ into the lumen, while alkali-metal cation/H^+^ antiporters are thought to recycle the extruded H^+^ in exchange for Na^+^ and K^+^^3^. Stellate cells appear to be the primary source of anion permeability, mainly chloride^1,2,4^ however less is known about the specific proteins involved in regulating chloride transport in these tissues.

Much of our knowledge concerning transepithelial chloride secretion in the MTs stems from research characterizing the diuretic effects of the leucokinin neuropeptides and the biogenic amine tyramine. Both tyramine and the leucokinins stimulate diuresis by increasing net chloride transport into the lumen^1,2,5^. They appear to act through distinct G-protein coupled receptors (GPCRs) that converge on the same second messenger pathway; the diuretic action of both secretory hormones is dependent on a rise in intracellular calcium levels specifically in stellate cells^2,6–8^. In contrast to the insect *Aedes*, where evidence points to a paracellular route for chloride secretion, in *Drosophila*, leucokinin stimulation activates a transcellular pathway for chloride flux^9–11^. A ClC chloride channel, *ClC-a*, is expressed in the basolateral and apical membranes of stellate cells and is required for the leucokinin-mediated increase in cytoplasmic chloride levels and secretion^6^. However, its role in chloride exit through the apical membrane is unclear.

Here we describe a role for a pentameric chloride channel encoded by the *secCl* (CG7589) gene in hormone-induced transepithelial chloride conductance. We previously identified *secCl* (CG7589) and *pHCl-2* (CG11340) as highly divergent putative anion-selective Cys-loop pLGIC subunits in *Drosophila melanogaster*^12^. We demonstrate that *secCl* forms a homomeric chloride channel that is open in the absence of any ligand and is expressed in the apical membrane of stellate cells. Moreover, loss of *secCl* eliminates the effects of diuretic hormones and has a penetrant lethal phenotype in adults, demonstrating that *secCl* plays a significant role in secretion. These results suggest that in *Drosophila* MTs, a member of the pentameric, cys-loop ligand-gated ion channel family mediates transcellular chloride secretion without direct interaction with a ligand.

## Results

### *secCl* forms a constitutively open homomeric channel

*secCl* (CG7589) belongs to a subfamily of divergent Cys-loop LGICs in the *Drosophila* genome that also comprises *pHCl-2* and *CG6927*^12–15^. These putative channel subunits are both specific to and conserved among arthropods.

**Figure 1:**
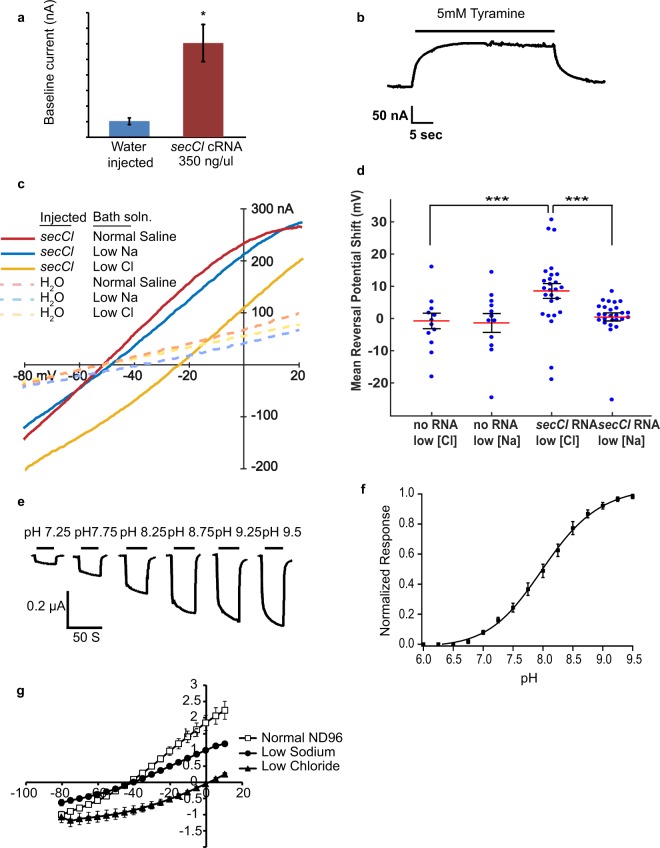
s*ecCl* forms a constitutively open homomeric chloride channel. (**a**) Bars indicate baseline currents of oocytes clamped at −80 mV that were injected with either water (n = 5) or *secCl* cRNA (n = 10). Error bars represent standard error of the mean. *P < 0.002 (two-tailed t-test). (**b**) Sample trace from an oocyte clamped at −80mV expressing *secCl*. The upward deflection corresponds to channels closing or being blocked upon treatment with 5 mM tyramine. (**c**) Sample current-voltage (IV) curves from an oocyte injected with *secCl* cRNA (solid traces) and a control oocyte injected with water (dashed traces). “Normal Saline” (ND96: 96 mM Na+ and 103.6 Cl^−^) “low Na” (6 mM Na^+^ and 103.6 mM Cl^−^) and”Low Cl” (96 mM Na^+^ and 13.6 mM Cl^−^). (**d**) Reversal potential shifts of *secCl*-expressing and control oocytes in low-chloride and low-sodium buffers relative to normal saline. ***P < 0.0001. (**e–g**) Coexpression of *secCl* with *CG6927* results in a heteromeric pH-sensitive chloride channel. (**e**) Representative traces from an oocyte clamped at −80 mV expressing *secCl* and *CG6927*, showing responses to changes in pH. Control buffer was ND96 at pH 6.0. (**f**) The response profile of the channel to changes in pH. The responses were normalized to the maximum current response of each oocyte. The curve represents the fit to the Hill equation (n = 5). (**g**) Representative traces of the current voltage relationship in “normal” (n = 6), “low sodium” (n = 4), and “low chloride” (n = 4) buffer. Error bars represent standard error of the mean.

To determine if *secCl* can form a functional channel, we isolated a *secCl* cDNA from wild-type flies that encodes a protein of 510 amino acids with the characteristic features of Cys-loop LGICs (Supplemental Figure S1) and no evidence of RNA editing^16^. We injected *Xenopus* oocytes with *secCl* cRNA and clamped the oocytes at −80 mV. Application of 1 mM acetylcholine, GABA, glutamate, glycine, histamine, serotonin, dopamine, nicotine, tyramine and octopamine to oocytes expressing *secCl* did not induce changes in holding current, indicating that none of these putative neurotransmitters gate *secCl* (data not shown). Even though *secCl* displays greatest homology to a class of pH-sensitive Cys-loop LGICs^14,15,17^, *secCl* -expressing oocytes were not sensitive to changes in pH (pH 6–pH 9.5). However, the holding currents of oocytes injected with *secCl* cRNA were significantly higher than water injected controls (302 nA ± 59.6 vs. 38.9 nA ± 10.7; p < 0.002, two tailed t-test) (Fig. 1a), indicating that an open ion channel is formed in *secCl* -expressing oocytes. Furthermore, exposure to 5 mM tyramine elicited a rapid and reversible, albeit modest, decrease in baseline current in *secCl* expressing oocytes (Fig. 1b), but had no effect on water injected oocytes. These results suggest that *secCl* forms a constitutively open ion channel that is weakly blocked by 5 mM tyramine.

*secCl* contains a motif that is highly predictive of chloride selectivity among Cys-loop LGICs^17–19^ (Supplemental Figure S1). In oocytes injected with *secCl* cRNA we observed a significant 8.5 ± 2.5 mV average positive shift in reversal potential in low-chloride buffer (13.6 mM Cl) relative to normal saline (103.6 mM Cl) compared to an insignificant 0.5 mV shift in low-sodium buffer (6 mM Na) relative to normal saline (96 mM Na) (Fig. 1c,d). In water-injected control oocytes the shifts were insignificant in both low chloride and low sodium buffers: −1.6 ± 2.5 mV (low Cl) and −1.3 ± 2.9 mV (low Cl). A reversal potential shift specific to low-chloride buffer is characteristic of chloride-selective channels. Although the mean and maximum (30.6 mV) shifts in reversal potential seen in *RNA*-injected oocytes were less than the 52 mV shift predicted by the Nernst equation for a chloride-selective channel, this was because: 1) not all RNA-injected oocytes were expressing *secCl*, and 2) we were unable to completely isolate *secCl* currents from endogenous oocyte currents that also contribute to the reversal potential.

Interestingly, *CG6927*, the third gene in the clade that includes *secCl* and *pHCl-2*, does not form a homomeric channel when expressed in oocytes but forms a pH sensitive, chloride-selective channel when co-expressed with *secCl* (Fig. 1d–f). Although *CG6927* is apparently not co-expressed with *secCl in vivo*^15,20^, the two subunits can form a heteromeric channel in oocytes that shows the expected shift in reversal potential (53.0 ± 1.5 mV) in low chloride buffer, supporting the conclusion that *secCl* is a functional chloride channel subunit.

### *secCl* is expressed in secretory tissues

**Figure 2:**
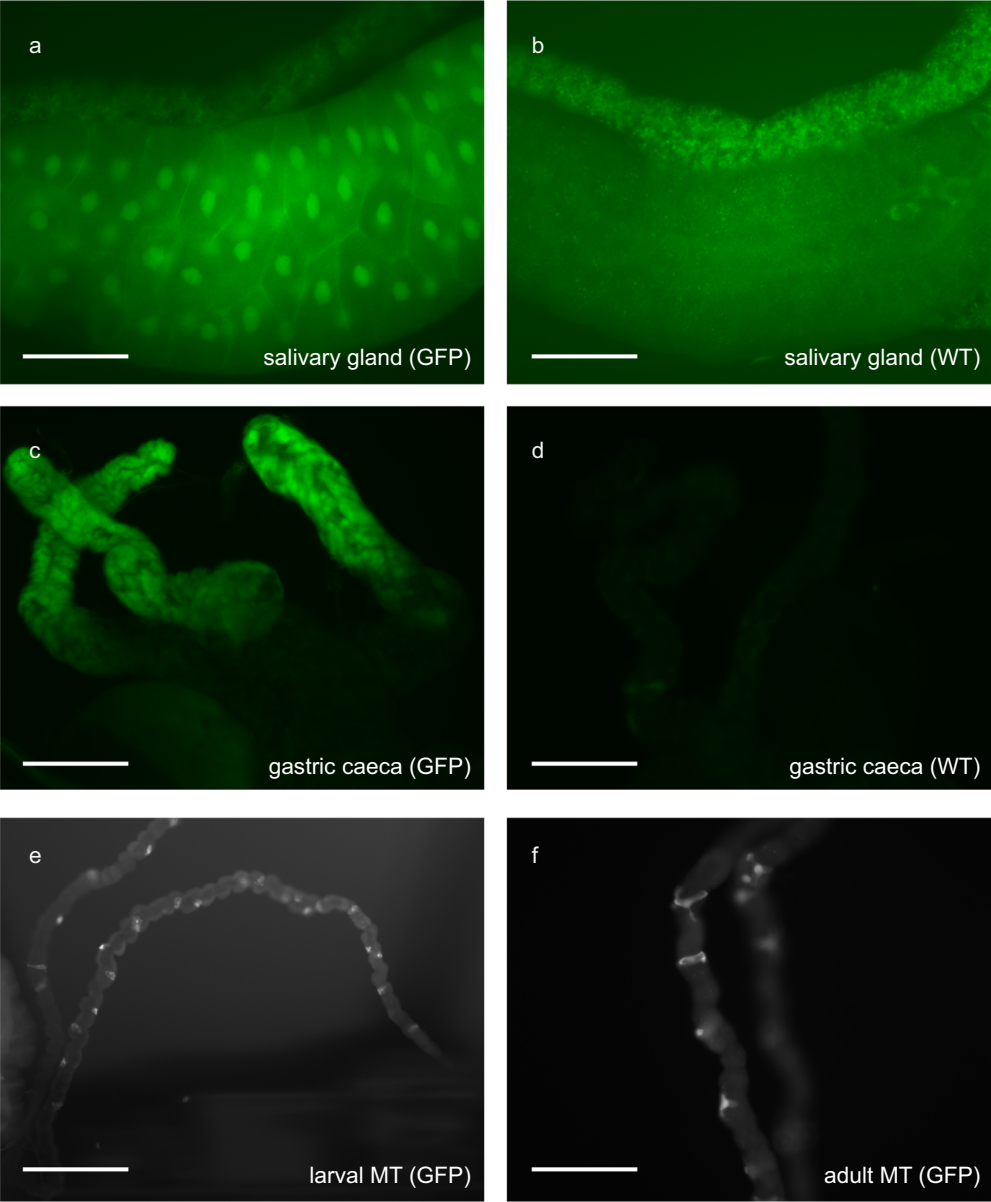
s*ecCl* gene expression patterns. Expression patterns of a *secCl* promoter-GFP fusion transgene. (**a–d**) Tissues of third instar larvae with (**a**,**c**; GFP) and without (**b,d**; WT) transgene. (**a–b**) Salivary glands. (**c**,**d**) Gastric cecae. (**e**,**f**) Main segments of MTs of third instar larvae (**e**) and adult (**f**) showing expression in stellate cells. Scale bars represent 100 μm.

Cys-loop LGICs are typically expressed in the nervous system and in muscle cells where they initiate rapid, ionotropic, post-synaptic communication in response to the presynaptic release of neurotransmitters^21^. In contrast, tissues imaged from flies bearing a *secCl* promoter-GFP construct showed fluorescence in the salivary glands and gastric caeca of third instar larvae (Fig. 2a–d), as well as in the stellate cells of initial/transitional and main segments of MTs in third instar larvae and adults (Fig. 2e,f), consistent with previous reports^15^. The GFP in the salivary glands localized to the nucleus for reasons that were unclear.

**Figure 3:**
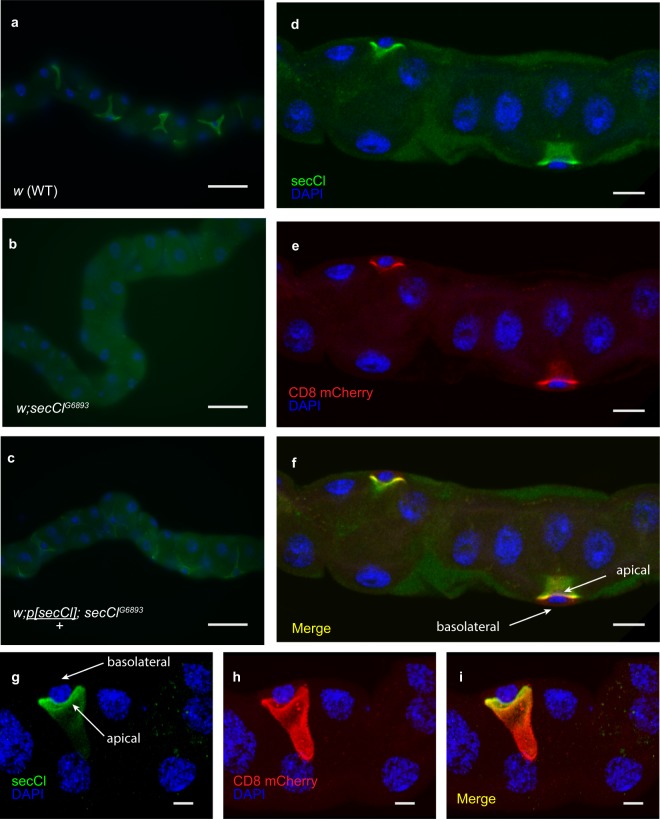
s*ecCl* protein is expressed in the apical membrane of stellate cells. Immunostaining of adult MTs using anti-*secCl* (green). Nuclei are labelled with DAPI (blue). (**a**) *secCl* is expressed in the stellate cells but not the principal cells of MTs. (**b**) Immunostaining is not observed in homozygous mutant *secCl*^*G689/G68933*^ MTs. (**c**) Immunostaining is restored to stellate cells in homozygous mutant *secCl*^*G6893/G6893*^ bearing a single copy of a wild-type *secCl* transgene, *p[secCl]*. (**d**) An optical cross-section of MTs stained with anti-*secCl* showing localization to apical membrane (luminal side of the nucleus). (**e**) Expression of the transgenic mCherry-tagged CD8 (red), which is a marker of the apical membrane in polarized epithelia. (**f**) Merge of (**d**) and (**e**) showing co-localization of *secCl* and CD8 (yellow). (**g–i**) A z-stack rendered in 3D and rotated showing single stellate cell. Colors as in (**d–f**). Scale bars in (**a–c**) represent 50 μm; (**d–f**) 10 μm; (**g–i**) 5 μm.

To determine the subcellular localization of *secCl* in polarized epithelia, we generated polyclonal antibodies raised against the unique intracellular loop. Immunostaining was detected exclusively in stellate cells of MTs (Fig. 3a). No immunostaining was detected in the MTs of homozygous *secCl*^*G6893*/*G6893*^ individuals (Fig. 3b), and the stellate cell-specific signal was restored in MTs from *secCl*^*G6893*/*G6893*^ homozygous mutants bearing a wild-type transgene for *secCl* (Fig. 3c), thus confirming that the signal observed in wild-type corresponds to *secCl* protein, and that *secCl* is dramatically reduced in the *secCl*^*G6893*/*G6893*^ homozygous mutants. Next we immunostained *secCl* in stellate cells expressing CD8-mCherry, a marker of apical membranes in polarized epithelia^22^. The *secCl* and CD8 staining were co-localized to the lumenal side of the nucleus (Fig. 3d–i), confirming that *secCl* is localized to the apical membrane in stellate cells.

### *secCl* regulates fluid secretion in the Malpighian tubules

**Figure 4:**
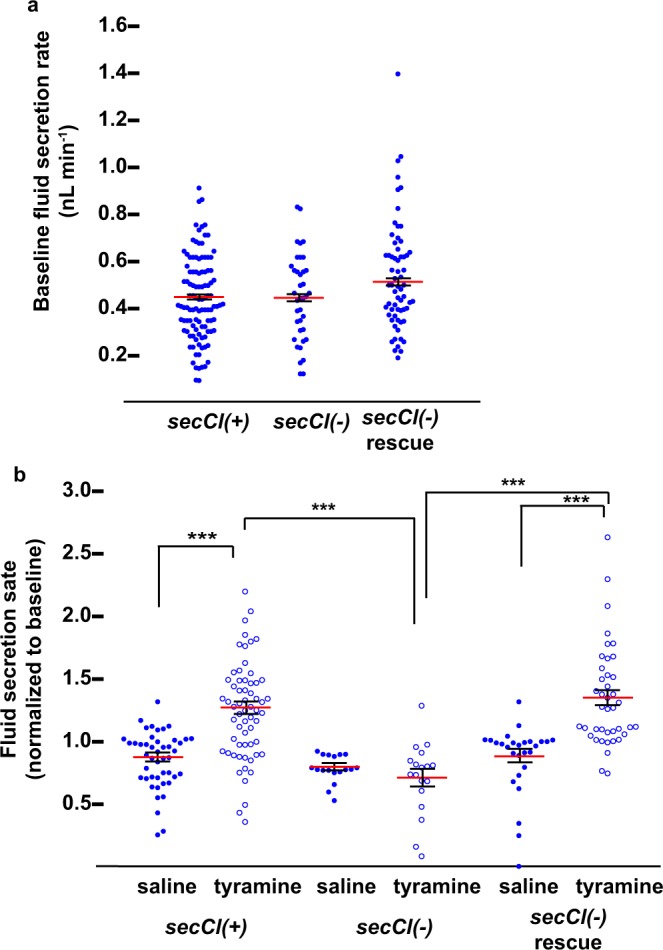
se*cCl* is required for the tyramine-mediated diuretic response. Fluid secretion assays conducted on tubules with the genotypes: *w (secCl(*+*))*, *w; secCl*^*G6893/G6893*^ *(secCl(*−*))* and *w; p[secCl], secCl*^*G6893/G6893*^ *(secCl(*−*) rescue)*. (**a**) Basal fluid secretion rate (FSR) in *Drosophila* saline^42^ over 40 minutes. (**b**) Change in FSR in response to 2.9 μM tyramine or *Drosophila* saline (mock treatment) normalized to the first 40 minute interval. Red horizontal bar indicates the mean and error bars represent standard error of the mean. *** indicates p < 10^−5^. Significance was estimated by ANOVA and pairwise comparisons by Tukey’s HSD.

The tissues where we found *secCl* expression, midgut, salivary glands and MTs, are all secretory tissues that rely on ion transport to facilitate digestion, generate saliva and regulate urine production respectively^23–25^. To determine if *secCl* is necessary for secretion, we used the Ramsey assay to determine fluid secretion rates (FSR) in the MTs^3^. We first measured the basal output of explanted MTs. In standard *Drosophila* saline, wild-type MTs spontaneously secreted fluid at 0.43 ± 0.02 nL min^−1^ compared to 0.48 ± 0.02 nl min^−1^ from *secCl*^*G6893/G6893*^ MTs, which lack *secCl* protein. Thus, loss of *secCl* does not impair basal FSRs (Fig. 4a).

We next asked whether *secCl* mutants affected diuretic stimulation of secretion. The biogenic amine tyramine and the neuropeptide leucokinin stimulate fluid secretion by increasing transepithelial chloride conductance, a process that is dependent on intracellular calcium signaling specifically in stellate cells^2,6,8,26^. Exposure to 2.9 μM tyramine increased FSRs in wild-type by 55.8 ± 7.93% compared to mock-treated controls, whereas the output of MTs from *secCl*^*G6893/G6893*^ loss-of-function mutants in response to tyramine were indistinguishable from mock-treated controls (Fig. 4b,c). Moreover, the diuretic effects of tyramine were restored in *secCl*^*G6893*/*G6893*^ MTs expressing a wild-type *secCl* transgene, as fluid secretion increased by 53.0 ± 6.48% in response to 2.9 μM tyramine compared to mock-treated controls (Fig. 4b,c). Thus, *secCl* is required for the diuretic response mediated by tyramine.

### *secCl* is necessary for chloride currents in response to the diuretic hormone tyramine

**Figure 5:**
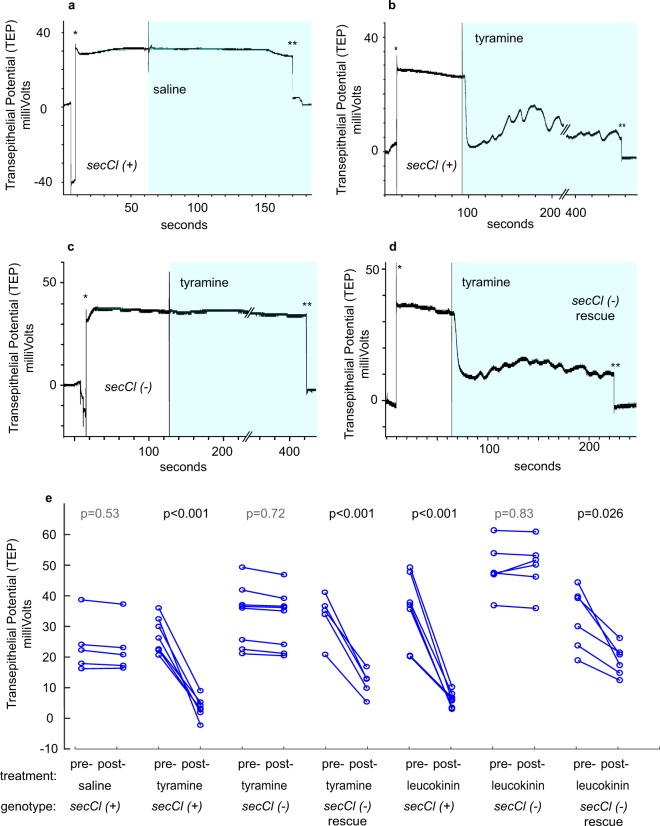
se*cCl* is necessary for the decrease in transepithelial potential in response to tyramine and leucokinin. Responses of the transepithelial potential (TEP) to tyramine. (**a–d**) Recordings of TEP in isolated Malpighian tubules. ‘*’ indicates penetration of the electrode into the lumen and ‘**’ indicates exit of electrode. The shaded area indicates the period of perfusion with saline or 2.9 μM tyramine as indicated. The beginning of perfusion was marked by a recording artifact. (**a**) The TEP of control *w* (*secCl(*+*)*) tubules did not respond to perfusion with saline. (**b**) In response to tyramine, the TEP of control tubules dropped to near zero mV and began to oscillate. (**c**) Tubules from w; *secCl*^*G6893/G6893*^ *(secCl(*−*))* flies did not respond to tyramine with a voltage drop. (**d**) Rescuing the *secCl*^*G689/G68933*^ with a wild-type transgene (*w; p[secCl]/*+; *secCl*^*G6893/G6893*^) restored the voltage drop and oscillations in response to tyramine. (**e**) P-values indicate the significance of the median change in absolute TEP pre- vs post-treatment (15 seconds after perfusion with saline control, 2.9 μM tyramine or 5 μM leucokinin) by Mann-Whitney U test.

In the resting state, the transepithelial potential (TEP) of the Drosophila Malpighian tubule is positive, reflecting the accumulation of positive charge in the lumen, ultimately attributable to the activity of a vacuolar-type proton ATPase (V-ATPase). Positive charge accumulates because the rate of flow of a negative counter ion, chloride, is limiting. In response to the diuretic hormones tyramine and leucokinin, the transcellular chloride conductance through the stellate cells increases. The increased chloride flux neutralizes positive charge in the lumen and collapses the TEP. Increased ion flux into the lumen in turn increases osmotically driven secretion. Absence of *secCl* could prevent diuretic-induced secretion by: 1) reducing activity of the V-ATPase such that chloride conductance is not limiting for secretion, or 2) by preventing the increase in chloride conductance in response to diuretic hormone. In the first case we would predict that the resting TEP would be reduced in the *secCl* mutant relative to wild type, whereas in the second case we would predict that the resting TEP would be normal (positive) but would fail to collapse in response to diuretic hormone. We compared TEP in wild type to *secCl*^*G6893*/*G6893*^ before and after treatment with 2.2 μM tyramine (Fig. 5a–e). Representative traces in Fig. 5 show the expected positive TEP prior to the exposure to tyramine and the collapse of the TEP to near zero potential after perfusion with tyramine. In contrast, the *secCl* loss-of-function mutant has a normal (positive) resting TEP but fails to depolarize upon perfusion with tyramine. We rescued the depolarization in response to tyramine by introducing the *secCl* transgene into the *secCl*^*G6893*/*G6893*^ background (Fig. 5d,e). We likewise observed that treatment with the peptide hormone leukokinin resulted in a consistent decrease in TEP in the rescued strain, but not in the *secCl*^*G6893*/*G6893*^ mutant. Thus, *secCl* is necessary for the increase in chloride conductance in response to diuretic hormones (Fig. 5e).

### Loss of *secCl* is lethal

We observed that the *secCl*^*G6893*^ allele was recessive and semi-lethal as the emergence of homozygous *secCl*^*G6893*/*G6893*^ adult progeny arising from heterozygous parents was 94% less than would be expected by Mendelian ratios (Table 1). Flies transheterozygous for *secCl*^*G6893*^ and a deficiency chromosome (*secCl*^*Df*^), which contains a deletion that spans over 100 kb and removes the entire *secCl* gene, display a similar degree of lethality (94%) as *secCl*^*G6893*/*G6893*^, suggesting that the *secCl*^*G6893*^ allele is null for *secCl* function. Moreover, introducing a single copy of a wild-type *secCl* transgene into the genetic background of *secCl*^*G6893*/*G6893*^ rescued 89–100% of the lethality, confirming that the lethal phenotype is due to loss of *secCl*. Restoring *secCl* specifically to stellate cells by using *c724-Gal4* to drive *UAS*::*secCl* in a *secCl*^*G6893*/*G6893*^ mutant background did not rescue the lethality (Table 1), indicating that *secCl* expression in the stellate cells of the MTs is not sufficient for viability.

**Table 1:** Lethal phenotype of *secCl*^*G6893*^ loss-of-function allele and *secCl* RNAi.

Genetic Cross:	# Of Homozygous Mutants Or RNAi (Obs/Exp) No Rescue ^1^	% Surv-IvaL^2^	# Of Homozygous Mutants (Obs/Exp) Rescued^1^	% Rescued Survival^3^
${\boldsymbol{yw}};\,\frac{{\boldsymbol{secC}}{{\boldsymbol{l}}}^{{\boldsymbol{G}}6893}}{{\boldsymbol{TM}}3}{\bf{x}}\,{\boldsymbol{yw}};\,\frac{{\boldsymbol{secC}}{{\boldsymbol{l}}}^{{\boldsymbol{G}}6893}}{{\boldsymbol{TM}}3}$	29/458	6%	—	—
${\boldsymbol{yw}};\,\frac{{\boldsymbol{secC}}{{\boldsymbol{l}}}^{{\boldsymbol{G}}6893}}{{\boldsymbol{TM}}3}{\bf{x}}\,{\boldsymbol{yw}};\,\frac{{\boldsymbol{secC}}{{\boldsymbol{l}}}^{{\boldsymbol{Df}}}}{{\boldsymbol{TM}}6}$	18/308	6%	—	—
${\boldsymbol{yw}};\frac{{\boldsymbol{secC}}{{\boldsymbol{l}}}^{{\boldsymbol{G}}6893}}{{\boldsymbol{TM}}3}{\bf{x}}\,{\boldsymbol{yw}};\,\frac{{\boldsymbol{P}}[{\boldsymbol{secCl}}]}{+};\frac{{\boldsymbol{secC}}{{\boldsymbol{l}}}^{{\boldsymbol{G}}6893}}{{\boldsymbol{TM}}6}$	47/235	20%	208/235	89%
${\boldsymbol{yw}};\,\frac{{\boldsymbol{secC}}{{\boldsymbol{l}}}^{{\boldsymbol{G}}6893}}{{\boldsymbol{TM}}3}{\bf{x}}\,{\boldsymbol{yw}};\frac{{\boldsymbol{P}}[{\boldsymbol{secCl}}]}{+};\,\frac{{\boldsymbol{secC}}{{\boldsymbol{l}}}^{{\boldsymbol{G}}6893}}{{\boldsymbol{TM}}3}$	37/243	16%	260/243	106%
${\boldsymbol{w}};\frac{{\boldsymbol{c}}724-{\boldsymbol{Gal}}4}{+};\,\frac{{\boldsymbol{secC}}{{\boldsymbol{l}}}^{{\boldsymbol{G}}6893}}{{\boldsymbol{TM}}3}{\bf{x}}\,{\boldsymbol{w}};\,\frac{{\boldsymbol{UAS}}-{\boldsymbol{secCl}}}{{\boldsymbol{Gla}}};\,\frac{{\boldsymbol{secC}}{{\boldsymbol{l}}}^{{\boldsymbol{G}}6893}}{{\boldsymbol{TM}}3}$	59/163	36%	13/54	24%
${\boldsymbol{w}};\,\frac{{\boldsymbol{CyO}}}{+};\,\frac{{\boldsymbol{UAS}}-\mathrm{secCl}\,\mathrm{RNAi}}{{\boldsymbol{TM}}3}{\bf{x}}\,{\boldsymbol{w}}/{\boldsymbol{Y}};\,\frac{{\boldsymbol{c}}724-{\boldsymbol{Gal}}4}{{\boldsymbol{S}}};\,\frac{{\boldsymbol{Pri}}}{+}$	119/104^4^	114%		

^1^The expected values are calculated from Mendelian ratios.

^2^Percent survival was calculated as the observed/expected ratio in column 2.

^3^Rescue efficiency was calculated as the observed/expected ratio in column 4.

^4^Viability after targeted knockdown of *SecCl* by RNAi driven by *c724-GAL4*. Expected survival is based on total progeny bearing the *UAS-secCl RNAi* transgene. Data also includes progeny from crosses*: w; GAL4/CyO; Pri/*+ *x w/Y; S/*+*; RNAi/TM3, Ser* and *w; S/*+*; RNAi/TM3, Ser x w/Y; GAL4/Cyo; Pri/*+.

### Loss of *secCl* in the MTs increases adult resistance to desiccation but not to salt stress

**Figure 6:**
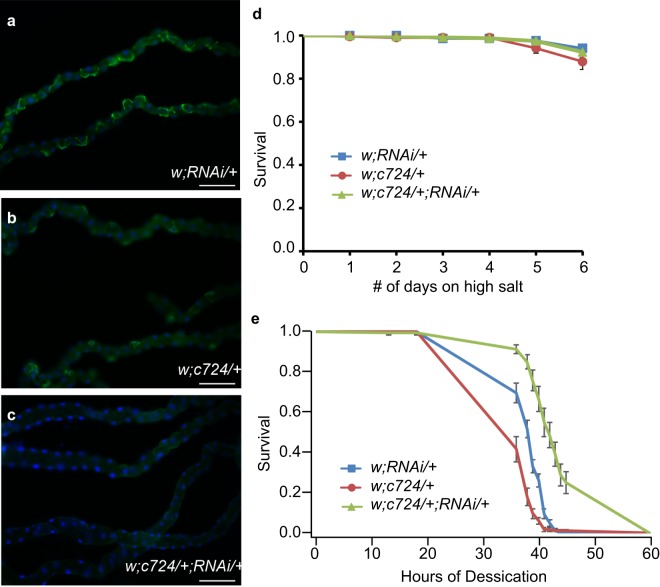
Genetic knockdown of *secCl* in stellate cells does not alter viability on a high-salt diet but does increase resistance to desiccation. (**a–c**) Immunostaining of adult MTs using anti-*secCl* (green). Nuclei are labelled with DAPI (blue). *secCl* expression is observed in MTs from undriven RNAi (**a**) and *c724-Gal4* (**b**) control lines. *secCl* protein is not detected in MTs where the RNAi is driven in stellate cells (**c**). (**d**) Survival rates of *w; RNAi/*+, *w; c724/*+ and *w; c724/*+; *RNAi/*+ maintained on a NaCl rich diet over six days. N = 11–12 vials of 20 flies (see methods) for each genotype. Survival rates of *w; RNAi/*+, *w; c724/*+ and *w; c724/*+; *RNAi/*+ under conditions of desiccation stress over the course of 60 hours. N = 11–14 vials of 10 flies (see methods) per genotype. Scale bars represent 100 μm. For (**d**) and (**e**), error bars represent standard error of the mean. For (**e**) survival rates for *w; c724/*+; *RNAi/*+ are significantly different from *w; RNAi/*+ and *w; c724/*+ at all points between18–45 hours (p < 0.002, two tailed t-test).

Because *secCl* expression in MTs was necessary for hormone-induced diuresis but not viability, we investigated the role of hormone-induced diuresis in *Drosophila* physiology. We hypothesized that diuresis is important for osmoregulation, which can be tested by raising flies on a salt-rich diet^27–29^. We used *c724-Gal4* to drive expression of a *UAS- secCl* -RNAi construct to knock down *secCl* specifically in stellate cells. Immunostaining showed that SecCl was expressed in MTs from control flies expressing either the *Gal4* or *UAS-RNAi* alone (Fig. 6a,b), but was undetectable in the MTs from individuals in which the RNAi was driven by the *Gal4* construct (Fig. 6c), thus verifying the efficacy of the knock down. Targeted knockdown of *secCl* exclusively in the stellate cells did not result in a reduction of adult viability (Table 1), nor did it affect adult survival on a diet containing 0.5 M extra NaCl (Fig. 6d), suggesting that loss of *secCl* in MTs does not compromise the osmoregulatory processes required to survive, even under conditions of high salt stress.

Since stimulated diuresis is reduced in *secCl* mutants, we hypothesized that removing *secCl* from the MTs might lead to a reduction in overall fluid loss, which would increase resistance to desiccation. To test this hypothesis, we compared the survival of adult flies with *secCl* RNAi knock-down in the stellate cells with the corresponding controls (Fig. 6e). Flies with *secCl* knocked down in the MT stellate cells exhibited significantly prolonged survival compared to controls under conditions of desiccation, suggesting that loss of *secCl* in the MTs increases resistance to desiccation.

## Discussion

The family of pentameric (Cys-loop) ligand-gated ion channels mediates fast, ionotropic neurotransmission in all bilateria studied. However, it is becoming increasingly clear that this family of ion channels has functions beyond ligand-gated neurotransmission. We previously showed that *pHCl-2*, which forms a proton gated anion channel, functions in the principal cells of MTs to modulate urine secretion^17^. Here we present evidence that a related channel subunit, *secCl*, forms a channel that mediates chloride flux in Malpighian tubules in response to diuretic hormones.

### *secCl* is required for hormone-mediated diuresis in the Malpighian tubules

Transepithelial chloride secretion in the MTs is generally thought to occur through or around stellate cells. Chloride channels have been identified in excised apical membrane patches of stellate cells and chloride conductance “hotspots” identified by vibrating probe analysis have been shown to localize at stellate cells^2^, suggesting a transcellular route for chloride secretion. The diuretic actions of tyramine and leukokinin are mediated by a rise in transepithelial chloride secretion in stellate cells^2,8,26^. Signalling by both hormones requires the coupled action of phospholipase C (PLC) and inositol triphosphate (IP_3_) to increase intracellular calcium levels in stellate cells^30^ and the two signalling pathways display cross-desensitization at the level of intracellular calcium signaling^5,31^. However, the specific link between intracellular calcium signalling and increased chloride conductance has remained elusive.

**Figure 7:**
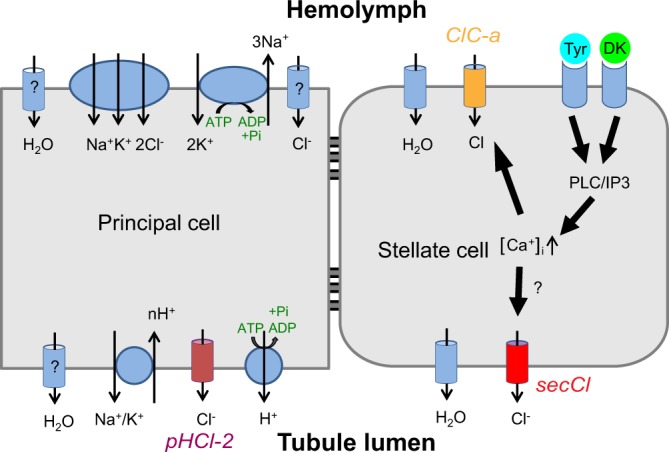
Model: The role of *secCl* within the tyramine/leucokinin diuretic pathway. A model describing the role of *secCl* within the signaling pathways of the diuretic hormones tyramine (Tyr) and *Drosophila* leucokinin (DK). Diuretic hormones bind to their respective GPCRs on the basolateral membrane of stellate cells, which triggers the release of calcium from intracellular stores via the PLC/IP3 pathway. Through an unknown mechanism, this rise in intracellular [Ca^+^^+^] results in the activation of *ClC-a* (orange), and *secCl* (red). ClC-*a*, localized to the basolateral membrane, provides necessary Cl^−^ entry into the cell from the hemolymph and *secCl*, localized to the apical membrane, provides a route for Cl^−^ exit into the lumen. FSRs increase as a consequence of increased chloride secretion.

We have shown that *secCl* expression is necessary for tyramine-mediated diuresis and that its expression in stellate cells is sufficient to restore diuresis in the mutant background. Thus, the *secCl* channel is a central element in the path from endocrine signalling to transepithelial chloride permeability (Fig. 7). Secretion in MTs is powered by an electrochemical proton gradient generated by a V-ATPase in principal cells. The lumen-positive apical electrochemical proton gradient is used to drive sodium and potassium transport into the lumen along with chloride counter ions. The resulting osmotic gradient drives fluid secretion. Basal FSR is limited by the chloride flux as indicated by: 1) the positive basal transepithelial potential, 2) the increase in chloride conductance when secretion is stimulated with diuretic hormones, and 3) the coincident collapse of the TEP to near zero potential as chloride conductance ceases to be limiting. Because TEP did not collapse in hormone-treated *secCl* mutants, we conclude that *secCl* is necessary specifically for the increase in chloride conductance. One possibility is that *secCl* is the chloride channel in the apical membrane of stellate cells that mediates the increase in chloride conductance in response to tyramine and leucokinin hormones.

*secCl* shares its hormone-induced secretion phenotype with another chloride channel, *ClC-a*, raising the question of what may be their respective roles^6^. Like *secCl*, *ClC-a* is expressed exclusively in stellate cells and is necessary in stellate cells for leucokinin-induced fluid secretion, a rise in intracellular chloride and TEP collapse. Unlike *secCl* however, *ClC-a* is localized to both the basolateral and apical plasma membranes with apparently greater accumulation at the basolateral membrane. These results are consistent with models in which: 1) *ClC-a* is necessary for *secCl* function, 2) *secCl* is necessary for *ClC-a* function, or 3) *secCl* and *ClC-a* act additively. The third model seems the least plausible based on biophysical principles; TEP should be highly sensitive to membrane conductance. Thus, if the *secCl* and *ClC-a* channels acted in parallel, in the absence of one channel we should see a change in TEP upon diuretic stimulation as a result of increased conductance through the other channel. Instead, we see no change in TEP in response to tyramine or leucokinin in the *secCl* mutant. The first two models appear equally plausible. Both pLGIC- and ClC-type channels have been implicated in vesicle trafficking and either could be responsible for correct trafficking of the other channel to the plasma membrane^32,33^. Alternatively, *ClC-a* may be primarily necessary for chloride entry into stellate cells via the basolateral membrane, whereas *secCl* is primarily necessary for the exit of chloride through the apical membrane. Thus, both channels would be necessary for transepithelial chloride flux, TEP collapse and diuresis.

In a model where *secCl* is the apical membrane chloride channel or is necessary for its expression, *secCl* could be constitutively active or co-regulated by the GPCR pathway that controls basolateral chloride influx in response to tyramine and leucokinin. For example, calcium-activated, protein kinase C-mediated phosphorylation of the intracellular loop is a known mechanism of pLGIC regulation^34^ that could gate the *secCl* channel or regulate its transport to the apical membrane.

### The *secCl*-mediated secretion in Drosophila physiology

*secCl* -mediated secretion is essential for viability as shown by the high lethality of flies lacking *secCl*. However, diuretic hormone-induced secretion in MTs is not necessary for viability in a laboratory setting, as *secCl* RNAi knock-down exclusively in stellate cells did not result in adult lethality (consistent with the viability of *ClC-a* mutants) nor did expression of *secCl* specifically in stellate cells rescue the lethality of the *secCl* mutant. Apparently *secCl*’s roles in the salivary glands and/or midgut are critical to viability, possibly because secretion in these tissues is essential for digestion. Instead, flies in which *secCl* was exclusively knocked down in stellate cells exhibited prolonged survival under conditions of desiccation, indicating that *secCl’s* role in hormone-induced diuresis is important for water balance.

### *secCl* is not primarily a neurotransmitter receptor

*secCl* was previously identified, along with *pHCl-2*, as a highly divergent Cys-loop pLGIC subunit that is arthropod specific^13,35^. Although relatively divergent, *secCl* has the structural characteristics of pLGIC neurotransmitter-gated channel subunits. However, we found that *secCl* forms a homomeric channel in *Xenopus* oocytes that is constitutively ion permeable in the absence of any obvious ligand. Moreover promoter-GFP expression (here and in^15^), immunocytochemistry and RNA expression data^20,36^ show that *secCl*, like *pHCl-2*, is primarily expressed in non-innervated, secretory tissues: salivary glands, gastric caeca of third instar larvae, as well as in stellate cells of larval and adult MTs. The gastric caeca and salivary glands are secretory tissues of the digestive system and the MTs are part of the excretory system. The *secCl-*expressing tissues do not receive synaptic input from the nervous system and we confirmed that *secCl* is localized to the apical membrane of MT stellate cells and therefore not directly exposed to hormones dispersed in haemolymph. Thus, *secCl* is unlikely to be directly gated by a neuroendocrine ligand.

### Is *secCl*’s role in secretion an ancestral function of pLGICs in metazoa?

*secCl* and the other channel subunits in its *Drosophila* clade, CG6927 and *pHCl-2*, are highly divergent relative not only to other pLGICs in *Drosophila* but also relative to the diverse pLGIC complements of other metazoan phyla: e.g. nematodes, molluscs and chordates^12,13,35^. Assuming that pLGIC subunits evolve at roughly equivalent rates, *secCl* and *pHCl-2* would thus represent an ancestral clade retained in *Drosphila* but lost in other phyla. pLGICs are present in eubacteria, archaebacteria and unicellular eukaryotes and therefore predate metazoa and the evolution of nervous systems^37,38^. We propose that *secCl* and *pHCl-2* may represent an ancestral function of pLGICs in metazoa prior to the evolution of complex nervous systems and the adaptation of pLGICs to their role as neurotransmitter receptors.

## Methods

### Cloning *secCl* cDNA

Whole RNA was purified from adult Oregon-R flies. First strand cDNA was synthesized using oligo (dT) primers and AMV (avian myeloblastosis virus) reverse transcriptase (Invitrogen). The *secCl* ORF was amplified by PCR (all primer sequences listed in Supplemental Data S2) and sub-cloned into the pDONR201 vector via the Gateway BP recombination system (Invitrogen) and verified by sequencing.

### *secCl* expression in Xenopus oocytes and electrophysiology

*secCl* cDNA was subcloned into a modified pT7 *Xenopus* expression vector using EcoRV and Xba1 cloning sites. The resulting *pT7-secCl* construct was linearized with BamH1 prior to synthesizing capped RNA (cRNA) by *in-vitro* transcription using the mMESSAGE mMACHINE T7 kit (Ambion). Oocytes were harvested from mature *Xenopus laevis* and injected with cRNA according to standard procedures^39^ and Two Electrode Voltage Clamp (TEVC) recordings were performed in ND96 buffer (‘Normal Saline’) as described in Feingold *et al*.^17^. For ion substitutions experiments, we substituted sodium acetate for sodium chloride (“Low Cl”) and cesium chloride for sodium chloride (“Low Na”). All animal care protocols comply with McGill’s animal care Standard Operating Procedures; protocol number 2006–5284. All animal care procedures were approved by the MGill Facility Animal Care Committee (FACC).

### Fly strains and maintenance

All *Drosophila* strains were maintained at room temperature and raised in standard cornmeal yeast sugar agar medium supplemented with dry yeast. As an apical membrane marker we used w*; *P{UAS-mCD8.ChRFP}2* (Bloomington Drosophila Stock Center (BDSC) # 27391) driven in stellate cells by the tissue specific driver *c724-Gal4* (a gift from Julian Dow)^40^. We used two *secCl* mutant backgrounds: the *secCl* deficiency, *w*^1118^; *Df(3* *L)Exel*1*6*1*32/TM6B,Tb* (BDSC) # 7611), and w*; *P{EP} secCl*^*G6893*/*G6893*^, which was derived from, *y*^1^,w*; p{EP} secCl^*G6893*^*/TM3,Sb*^*1*^*,Ser*^*1*^ (BDSC # 27218) and has a P-element inserted in the first intron of *secCl*. To achieve tissue-specific knockdown of *secCl*, we used *c724-Gal4* to drive w*; *P{TRIP.JF02437}attP2*, a U*AS- secCl* -RNAi strain generated from *y*^*1*^*,v*^*1*^*; P{TRIP.JF02437}attP2* (BDSC #27090).

A *secCl* rescue transgene, *P[secCl]* was generated by PCR-amplification of the *secCl* locus, including the *secCl* gene as well as flanking sequence spanning 2 kb upstream and 1 kb downstream of the open reading frame, from Oregon-R genomic DNA, followed by subcloning into the pENTR-SD-TOPO vector (Invitrogen) and recombination via Gateway LR into the pTWH destination vector (Drosophila Genomics Resource Center, DGRC), which contains sequences for P-element mediated transgenesis. Transgenic *w* flies expressing *p[secCl]* were generated by BestGene Inc. (www.thebestgene.com, California) and an insertion that mapped to chromosome 2 was used for all experiments. To drive green fluorescent protein (GFP) expression from the *secCl* promoter, a genomic region including 2 kb of upstream DNA and the first exon of *secCl* was amplified and cloned into the pTWG destination vector (DGRC) by LR recombination using the intermediate pENTR-TOPO-SD Gateway system. Mutation of two ATG sites prevent translation of the in-frame *secCl* signal sequence. Transgenics were generated by BestGene Inc., as above. For tissue specific expression under the GAL4/UAS system, the *secCl cDNA* was cloned downstream of the UAS promoter in the pTWH vector (DGRC, not HA-tagged), and the resulting construct was injected into *yw* embryos^41^.

### Immunohistochemistry

To raise polyclonal antibodies, the M3-M4 intracellular loop of *secCl cDNA* was amplified and the PCR product subcloned into the pEColi-Nterm 6xHN vector using the In-Fusion kit (Clontech). The peptide was expressed in BL21 cells and purified using Talon His-affinity binding columns (Clontech) and used to immunize rats. Antiserum was pre-absorbed to homozygous *secCl*^*G6893*^ third instar larvae fixed in 4% (vol vol−1) formaldehyde/PBS and used at a final concentration of 1:1000. antiRat-Alexa Fluor 488 secondary antibody (Invitrogen) was pre-absorbed to fixed wild-type (Oregon-R) embryos and used at a concentration of 1:1000. Immunostaining was performed as described in Feingold *et al*.^17^.

### Malpighian tubule dissection and Ramsay fluid secretion assays

MT dissections and Ramsay fluid secretion assays were carried out as described^2,42,43^ on adult females 3–10 days post eclosion. 1.5 minutes prior to the end of the first 40-minute interval, the bathing droplet (18 μl) was spiked with 2 μl *Drosophila* saline or 29 μM tyramine in *Drosophila* saline.

### Viability assays on high salt diet

Flies were raised on a standard diet except water was replaced with a 0.5 M NaCl solution as described by Huang *et al*.^27^. Viability of flies in vials with high-salt food was scored every 24 hours for 6 days. On day 3, flies were transferred into vials containing fresh, high-salt food.

### Desiccation assays

Adult females 3–10 days post eclosion were placed in empty vials in groups of 10 individuals. Survival was scored at time intervals, as indicated, until 100% mortality was reached.

### Transepithelial potential assays

Malpighian tubules from adult females 3–10 days post eclosion were dissected in *Drosophila* saline and mounted on glass coverslips coated with 100 μg/ml poly-L lysine. A sharp electrode (R = 20–40 MΩ) filled with 3 M KCl pulled from theta septum borosilicate glass (Warner Instruments, Hamden, CT, USA) was used to impale the tubule using a PCS-5000 micromanipulator (EXFO Burleigh, Mississauga, ON, Canada)^5^. Only tubules with TEP > +20 mV were used. An Axopatch 200 A amplifier, a CV 201 A headstage and pClamp 8 software (Axon Instruments, Sunnyvale, CA, USA) were used to record TEPs. 50 μl of 29 μM tyramine (Sigma-Aldrich) in saline or saline alone was then added to the 600 μl bath to achieve a concentration of 2.2 μM. Similarly, leukokinin I (Sigma-Aldrich) was diluted from a 65 μM stock solution. The electrode was removed from the lumen to ensure that the baseline drift was <3 mV.

### Statistical analysis

All data are presented as the mean ± SEM. Data were analyzed by two-tailed t-test in Igor-Pro (Wavemetrics) and ANOVA (Matlab, MathWorks).

## Supplementary information

Supplemental Data

## Data Availability

The datasets generated during the current study are available from the corresponding author on reasonable request.
