# Supplementary material for: secCl is a cys-loop ion channel necessary for the chloride conductance that mediates hormone-induced fluid secretion in Drosophila
Source: Sci Rep. 2019 May 16;9:7464. doi: 10.1038/s41598-019-42849-9 (PMC6522505; doi:10.1038/s41598-019-42849-9)
Supplement: Supplementary file 1 — Supplemental Data [file 41598_2019_42849_MOESM1_ESM.pdf]

Supplemental Information for:

secCl is a cys-loop ion channel necessary for the chloride conductance that mediates hormone-induced fluid secretion in *Drosophila*.

Daniel Feingold[1], Laura Knogler[4,5], Tanja Starc[2], Pierre Drapeau[4], Michael J. O'Donnell[3], Laura A. Nilson[1], and Joseph A. Dent[1]

1. Department of Biology, McGill University, 1205 Dr. Penfield, Montréal, Québec, Canada, H3A 1B1.
2. Institute of Neuroscience, Technische Universität München, Biedersteiner Str. 29, München, Germany, Bau 601D-80802.
3. Department of Biology, McMaster University, 1280 Main Street West, Hamilton, Ontario, Canada, L8S 4K1.
4. Department of Neurosciences, Research Centre of the University of Montréal Hospital Centre, Montréal, Québec, Canada
5. Max Planck Institute of Neurobiology, Sensorimotor Control Research Group, Am Klopferspitz 18, Martinsried 82152, Germany

<u>Running Title</u>: a cys-loop channel mediates hormone-induced diuresis.

```
secCl     1  -MNLHLSEIFWITLILVLSIWTSVGVKAEEEE---------CPSMADAGS-----LQQTQ   45
glc-1     1  MATWIVGKLIIASLILGIQAQQARTKSQDIFEDDNDNGTTTLESLARLTSPIHIPIEQPQ   60

secCl    46  LIQR--LTHVCR--YDRLERPFEYDSDGKRLPIVVKTRIYVYFLQNLNSDLLQFKMHALL  101
glc-1    61  TSDSKILAHLFTSGYDFRVRPPTDN--GG--PVVVSVNMLLRTISKIDVVNMEYSAQLTL  116

secCl   102  QLRFQDKRLAYKAFNRSDNILGQKHLSERLWLPHIFFANERESSILGTDEKDVLTSLSPE  161
glc-1   117  RESWIDKRLSYGVKGDGQPDFVILTVGHQIWMPDTFFPNEKQAYKHTIDKPNVLIRIHND  176

secCl   162  GNVIISTRMQASLYCWMNFQKFPFDQQFCSTVLESWMYNTSDLILEWEPHTPISFDPEMR  221
glc-1   177  GTVLYSVRISLVLSCPMYLQYYPMDVQQCSIDLASYAYTTKDIEYLWKEHSPLQLKVGLS  236
                                                                   M1
secCl   222  LTEYNMAQFWHNTTIVQSDGDNLRHGAFAGNYSSLSFTVNLKREIGFYLLDYYLPSMMIV  281
glc-1   237  SSLPSFQLTNTSTTYCTSVTN-------TGIYSCLRTTIQLKREFSFYLLQLYIPSCMLV  289
                                     M2
secCl   282  AISWVSFWLQADASPPRIMLGTSTMLSFITLSSSQSKNLPKVSYIKVSEVWFLGCTFFIF  341
glc-1   290  IVSWVSFWFDRTAIPARVTLGVTTLLTMTAQSAGINSQLPPVSYIKAIDVWIGACMTFIF  349
                 M3
secCl   342  GSLVEFAFVNTIWRRKENIELKKVNSKYIIKSTLTPRPARRQIGGSLSNESRARSCSSLD  401
glc-1   350  CALLEFALVNHIAN-KQGVERKARTER-----EKAEIPLLQNLHNDVP--------T---  392

secCl   402  NIVSSTESVRNGNGTVNQGFNNYLTVHPNLPIIRTECAEADTVSICSARTNNDHIVDVDK  461
glc-1   393  KVFNQEEKVR------------------TVPLNR--------------RQMNSFLNLLE-  419
                                  M4
secCl   462  DKKDTPPTFTTMTPQEIAMWIDRRSRFLFPAMFLAFNALYWTFVY---VL-          508
glc-1   420  --------T-KTEWNDISKRVDLISRALFPVLFFVFNILYWSRFGQQNVLF          461
```

Supplemental Figure S1: ***secCl* encodes a protein that contains the features of a typical cys-loop pentameric ligand-gated ion channel (pLGIC) subunit.** Amino acid allignment of *secCl* with the *Caenorhabditis elegans* pLGIC subunit *glc-1*. The two cysteines that form the cys-loop are boxed in red. The alpha-helical transmembrane domains M1-M4 are marked with solid green lines. Shading indicates degree of conservation (darker is more conserved) as scored by BLOSUM26 substitution matrix (Henikoff, S. & Henikoff, J.G. Amino Acid Substitution Matrices from Protein Blocks. *Proc. Natl. Acad. Sci. U. S. A.* **89**, 10915–10919 (1992)). Sequences aligned using CLUSTAL W (Thompson, J.D., Higgins, D.G. & Gibson, T.J. CLUSTAL W: improving the sensitivity of progressive multiple sequence alignment through sequence weighting, positions-specific gap penalties and weight matrix choice. *Nucleic Acids Res*, **22**,4673-4680 (1994).)

Supplemental Data S2

Primers for Amplification of *secCl* cDNA from mRNA:
5'GCGGGGACAAGTTTGTACAAAAAAGCAGGCTCGATATCATGAATCTGCATCTGAGTGA3'
5'GGGGACCACTTTGTACAAGAAAGCTGGGTGAGCTCTAGATTACAAAACATAGACGAGGT3'.

Primers for amplification of *secCl* genomic rescuing fragment:
5'CACCAATCGACGCAGGTAAATGGAC 3' and
5'AGGATCGAGGATAGACGCATGGTT3'.

Primers for amplification of *secCl* promoter for *secCl*-promoter::GFP fusion:
5'-CACCAATCGACGCAGGTAAATGGAC -3' and
5'-TCTTCCTCGGCTTTGACCCTA-3'

The primers used to introduce site specific mutations to remove extraneous start codons from the promoter:
5'-GCAGATTCCTCAGcTGGcTTGATTC-3' and 5'-TTGGATCACGCTGATCTTGGTC-3' where lower case letters refer to the nucleotide bases that were mutated.

Primers for amplification of *secCl* intracellular loop for antibody production:
5'TAAGGCCTCTGTCGACCAGTTTGCCTTTGTCAACAC3'
5'CAGAATTCGCAAGCTTCTACGGCGTGTCCTTTTTATCCT-3'
Nucleotides 1-16 of both primers correspond to homologous portions of the pEcoli- Nterm 6xHN vector.
